# Branching morphology determines signal propagation dynamics in neurons

**DOI:** 10.1038/s41598-017-09184-3

**Published:** 2017-08-21

**Authors:** Netanel Ofer, Orit Shefi, Gur Yaari

**Affiliations:** 1 Faculty of Engineering, Bar Ilan University, Ramat Gan, 5290002 Israel; 2 Bar Ilan Institute of Nanotechnologies and Advanced Materials, Bar Ilan University, Ramat Gan, 5290002 Israel

## Abstract

Computational modeling of signal propagation in neurons is critical to our understanding of basic principles underlying brain organization and activity. Exploring these models is used to address basic neuroscience questions as well as to gain insights for clinical applications. The seminal Hodgkin Huxley model is a common theoretical framework to study brain activity. It was mainly used to investigate the electrochemical and physical properties of neurons. The influence of neuronal structure on activity patterns was explored, however, the rich dynamics observed in neurons with different morphologies is not yet fully understood. Here, we study signal propagation in fundamental building blocks of neuronal branching trees, unbranched and branched axons. We show how these simple axonal elements can code information on spike trains, and how asymmetric responses can emerge in axonal branching points. This asymmetric phenomenon has been observed experimentally but until now lacked theoretical characterization. Together, our results suggest that axonal morphological parameters are instrumental in activity modulation and information coding. The insights gained from this work lay the ground for better understanding the interplay between function and form in real-world complex systems. It may also supply theoretical basis for the development of novel therapeutic approaches to damaged nervous systems.

## Introduction

Deciphering neuronal electrical activity and information flow in the brain is a great challenge in neuroscience^1–3^. Neurons are interconnected via their dendritic and axonal branching trees presenting complex morphologies. Studying the influence of these morphologies on electrical activity modulations is of crucial importance for understanding brain functionality. A fundamental building block of the neuronal branching tree is the branching point where a mother branch bifurcates into daughter branches. Previous studies raised the possibility that modulations in the frequency of action potential trains can be the result of spike failures along the axon and through the branching points^4–9^. Rall analyzed symmetric branching points in dendritic trees by the ‘equivalent cylinder’ approach, and then expanded the analysis to include various tree structures with passive and active membranes^10, 11^. He found the optimal diameter ratio between the mother and daughter branches which gives an impedance matching, and defined the geometric ratio (GR)^12, 13^:

1 $$GR=\frac{{\sum }_{j}{d}_{j}^{3/2}}{{d}_{a}^{3/2}},$$

where d_a_ is the diameter of the mother branch, and d_j_ are the diameters of the daughter branches. It was shown that the response in the daughter branches depends only on GR, and that the two daughter branches react identically, even for branches with different radii. For GR = 1, there is a perfect impedance match and action potentials smoothly cross the branching points. For GR < 1, action potentials cross the branching points with slight changes in shape and velocity, and for GR > 1 action potentials cross with a delay; the delay scales exponentially with GR^14^. For GR above a critical value that depends on the temperature action potentials fail to cross, leading to a blockage^15–17^. For high GRs that still allow propagation there are cases of reflection, where one spike continues to propagate into the daughter branches while another spike reverses up to the mother branch^18–20^. This ‘reflection spike’ may collide and annihilate the next spike^13, 21^.

Khodorov *et al*. and Parnas *et al*. extended the analysis of activity across branching points to several spike series^22–24^. Effects of complex axonal geometries on trains were described in numerous simulation studies^25–32^. Weaver and Wearne have shown that the ratio between axonal radius and length influences neuronal firing^33^. In a previous paper, we have shown the generation of firing patterns consisting of tunable number of action potentials combined with failures as a result of stimulus current and axonal segment geometry^34^. However, the effects of geometry of unbranched and branched axonal segments on activity still calls for further study.

The influence of the axonal morphology on activity was also demonstrated in experimental studies. Spira *et al*. recorded changes of spike train patterns, such as complete conduction block and intermittent failures at specific regions along the giant axon of the cockroach^35^. Ramon *et al*. have shown action potential modifications at sites of abrupt increase in axonal diameter^36, 37^. For high frequency current modulations in unbranched axons, patterns such as fragmented trains, quasi-periodic, and chaotic responses were observed^38–41^. Measurements along axonal branching points with two different radii exhibited different responses in the two daughter branches. In some cases the conduction block appeared first at one of the branches^42–46^, while in other experiments the conduction block occurred simultaneously in both daughter branches^47^. Stockbridge have shown that in branching points consist of short and long daughter branches, only the first of adjacent spike pair invades the long branch, while the two spikes propagate the short one^48, 49^. Sasaki *et al*. have examined changes in action potential width caused by modulations of axonal length and branching order^50, 51^. Differential modulation response between axonal branches was recorded using high spatio-temporal multi-electrode arrays^52–54^. The above experimental observations were explained using ad hoc theoretical arguments mainly involving extracellular factors, necessitate a unified view of the link between geometry and activity pattern formation.

Here we systematically study signal propagation in unbranched and branched axons by scanning stimulus frequencies and morphological parameters. We show how even simple axonal elements can code information on spike trains, and how asymmetric responses emerge in axonal branching points.

## Results

**Figure 1:**
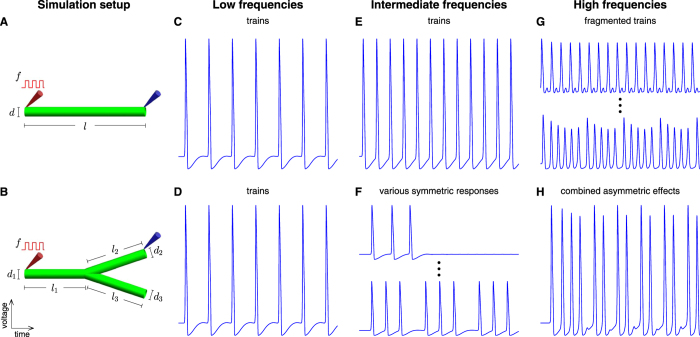
Representative responses to current stimuli along unbranched and branched axons. (**A**,**B**) Schematic diagrams of the studied axonal setups. The varied parameters are shown: stimulating frequency (*f*), segment diameter (*d*) and segment length (*l*). Stimulation and measure probes are indicated in red and blue arrows respectively. Representative responses in unbranched axons are shown for low- (**C**), intermediate- (**E**), and high- (**G**) frequency stimulations, while for branched axons the corresponding responses are shown in (**D**), (**F**) and (**H**).

To study the effects of geometry on electrophysiological activity we focused on the two fundamental blocks building the neuronal branching tree, axonal linear segments and axonal branching points. A spatial extension of the Hodgkin Huxley model was used to systematically explore the effects of geometrical parameters on activity patterns. Action potential trains were induced at the edges and were measured at multiple points along the segments (Fig. 1A,B). Response patterns were recorded for different stimulus frequencies in a wide range of radii and lengths. For low frequencies action potential trains propagate with no modification. As frequency increases, modulations appear in branching points. For higher frequencies, modulations also appear in unbranched segments, leading to asymmetric response in branched axons.

Figure 1 shows schematically the various behaviors observed by the two axonal elements. Note that the type of modulation is determined by geometry and is described in details in the sections below. In unbranched linear axons low frequency spike trains propagate uninterruptedly (Fig. 1C,E). For higher stimulus frequencies (>146 Hz, for the parameters specified in Supplementary Table S1), failures occur leading to modulated fragmented trains (Fig. 1G). In branched axons, modulations in spike trains occur already at lower frequencies. At those intermediate frequencies (51–146 Hz, for the same parameters) the firing pattern is symmetric between the two daughter branches even in geometrically asymmetric branching points (Fig. 1F). At higher frequencies, combined effects from the unbranched and branched behavior repertoires are generated leading to asymmetric firing patterns between the two daughter branches (Fig. 1H). Frequency values separating the different regimes depend on other parameters of the model. The frequency values indicated above refer to model parameters described in methods section.

## Characterization of responses along unbranched axon

**Figure 2:**
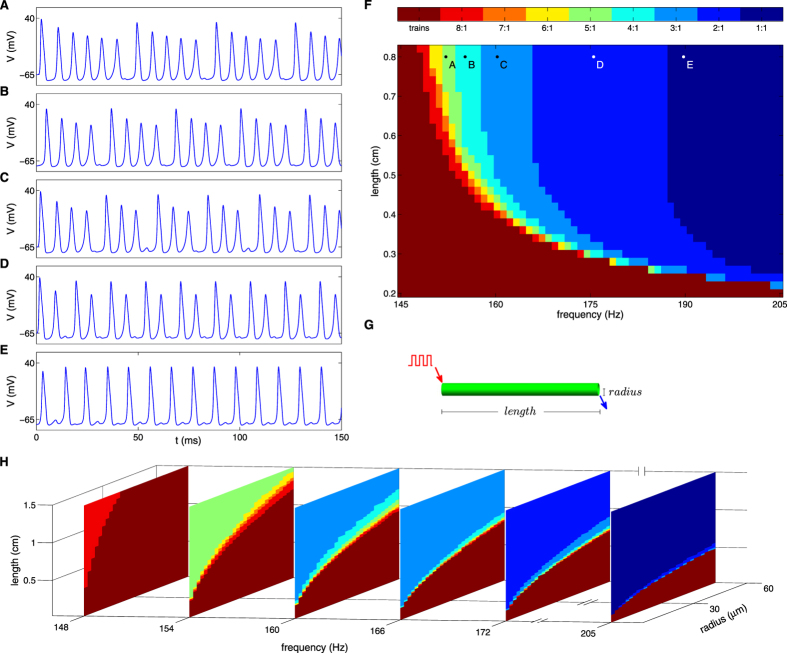
Responses to current stimuli in the studied phase space for unbranched axons. (**A**–**E**) Time dynamics of propagating signals generated with parameters indicated in phase plane diagram (**F**). The colors in the phase plane diagram represent different firing patterns as shown in the color key. The axonal radius used to construct the phase plane is 10 µm, and the signals were measured as schematically shown in (**G**) by a blue arrow. (**H**) Z-stack phase plane diagrams of axonal radius vs. axonal length are shown for six stimulus frequencies. Same color code as in (**F**).

For the range of frequencies studied here, all pulses at the stimulation point generate spikes. A fraction of these spikes fail to propagate leading to a ‘fragmented train’ pattern. In Fig. 2A–E examples of ‘fragmented trains’ are shown. As stimulus frequency increases a larger fraction of spikes fail to propagate. Figure 2F is a phase plane diagram summarizing firing patterns as a function of axonal length and stimulus frequency. The axonal length of the transition between trains that fully propagate (color coded in brown) and trains with failures (color coded in red) monotonically decreases with stimulation frequency, and its functional form is derived below. The response for higher stimulus frequencies can be seen in Supplementary Fig. S1. In this regime (>300 Hz for length >0.5 cm), a single spike followed by several failures is propagated. For yet higher frequencies (>440 Hz for length >0.42 cm), only a finite number of spikes succeed to propagate, followed by a flat signal. To better understand axonal radius role, the studied phase plane was extended to three dimensions including radius parameter (Fig. 2H). It can be seen that the axonal length of the transition between trains that fully propagate and trains with failures monotonically increases with radius.

Fragmented trains emerge along the axon via a two stage process. First, single spike failure events occur. Next, spikes are shifted in time to yield a lower frequency train pattern with equivalent intervals between spikes. Supplementary Fig. S2 demonstrates this process by showing the propagating signal at multiple probes along the axon for a stimulus frequency of 160 Hz. At stimulation point, all current pulses generate corresponding spikes (Supp. Fig. S2B). At a close proximity to the stimulation point, the amplitude of spikes is modulated, and a four spike periodic pattern appears (Supp. Fig. S2C). As spike train travels along the axon, the lowest spike of each group diminishes (Supp. Fig. S2D). Further down the axon, spikes amplitude and inter-spike intervals are equalized (Supp. Fig. S2E,F), resulting in a 120 Hz spike train regular pattern.

### Functional form of the critical axonal length separating uninterrupted and fragmented trains

**Figure 3:**
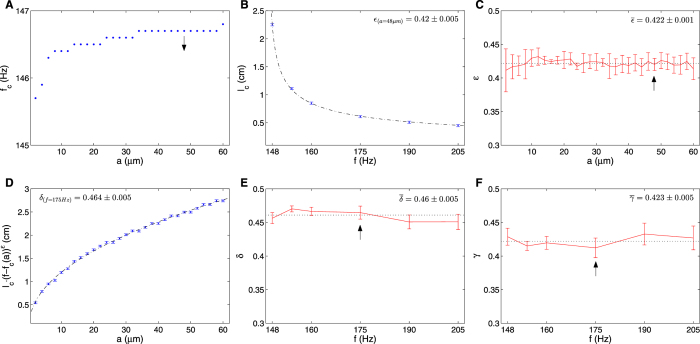
Parameter estimation of the phenomenological equation that describes the transition between propagating train and coded firing patterns. (**A**) Direct measurements of the maximum frequency (*f*_*c*_) that enables propagation of uninterrupted train in long axons (10 cm) for a range of axonal radii. Arrow indicates radius value used in (**B**). (**B**) Fitting the dependency between *l*_*c*_ and *f* for *a* = 48 µm. Dashed line is the fitted curve: $2.517\cdot {(f-146.7)}^{-0.42}$. Bars correspond to numerical measurement errors determined by the sampling interval of the phase plain: *Δl* = *0.02* *cm*. (**C**) Fitted ε’s for all studied radii. Arrow indicates ε value calculated in (**B**). (**D**) Fitting the dependency between ${l}_{c}\cdot {(f-{f}_{c}(a))}^{\varepsilon }$ and *a* for *f* = 175 Hz. Dashed line is the fitted curve: $0.4122\cdot {(f-{f}_{c}(a))}^{-\varepsilon }\cdot {a}^{0.464}$. Bars correspond to numerical measurement errors determined by the sampling interval of the phase plain: *Δl* = *0.02* *cm*. (**E**,**F**) Fitted δ’s (**E**) and γ’s (**F**) for all studied frequencies. Arrows indicate the δ and γ values calculated in (**D**).

Figure 2F and H suggest a relatively simple dependency between stimulation frequency and axonal radius, and the critical axonal length separating uninterrupted and fragmented trains (the borders of the brown regions). The simplest functional form that describes reliably this transition is a product between a power law dependency for the radius and a shifted power law dependency for the frequency:$\,{l}_{c}(a,f)=\gamma \cdot {a}^{\delta }\cdot {(f-{f}_{c}(a))}^{-\varepsilon }$, where *l*_*c*_ is the transition critical length, *a* is the axonal radius, *f* is the train frequency, and *f*_*c*_ is the maximum frequency that enables propagation of uninterrupted train in long axons. Directly measuring *f*_*c*_ for a range of axonal radii revealed a dependency on this parameter (*f*_*c*_ *(a)*, see Fig. 3A). Knowing *f*_*c*_ *(a*), we sought to estimate the free parameters $(\gamma ,\delta ,\varepsilon )$. To estimate $\varepsilon$, we fixed the value of *a* and fitted the dependency between *l*_*c*_ and *f*. An example for this fit is shown in Fig. 3B for *a* = 48 µm (indicated by arrows in Fig. 3A and C). $\varepsilon$ estimations for 30 axonal radii were averaged to yield a combined estimate of ε = 0.422 ± 0.001 (Fig. 3C). Having $\varepsilon$ and *f*_*c*_ *(a*) in hand, allowed us to estimate $\delta$ and $\gamma$ by fitting the dependency between ${l}_{c}\cdot {(f-{f}_{c}(a))}^{\varepsilon }$ and $a$ for a given frequency. An example for this fit is shown in Fig. 3D for *f* = 175 Hz (indicated by arrows in Fig. 3E and F). $\delta$ and $\gamma$ estimations for 6 frequencies were averaged to yield combined estimates of $\delta$ = 0.46 ± 0.005 (Fig. 3E), and $\gamma$ = 0.423 ± 0.005 (Fig. 3F). These estimates yield the following equation:

2 $${l}_{c}(a,f)=0.423\frac{{a}^{0.46}}{{(f-{f}_{c}(a))}^{0.422}}$$

Analogous analysis for higher temperature (20 °C) could be found in Supplementary Note S1 yielding a similar expression.

## Characterization of responses along branched axons

### Responses in symmetric branched axons

**Figure 4:**
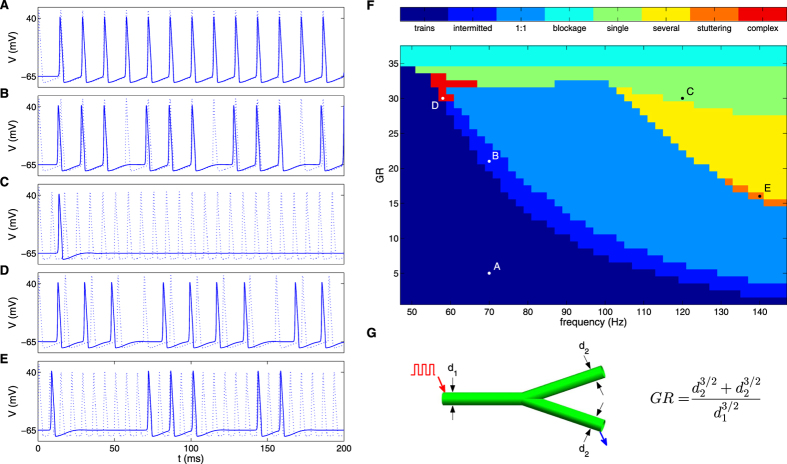
Responses to current stimuli in the studied phase space for branched axons. (**A**–**E**) time dynamics of propagating signals generated with parameters indicated in phase plane diagram (**F**), the dashed line indicates the response at the stimulation point near the beginning of the mother branch. The colors in the phase plane diagram represent different firing patterns as shown in the color key. The axonal radius of the mother branch used to construct the phase plane is 10 µm, each segment is 2 cm, and the signals were measured as schematically shown in (**G**) by a blue arrow.

To explore the influence of axonal branching point on information flow, we studied propagation dynamics along branched axons composed of trunks that bifurcate into daughter branches with identical radii. This setup allowed us to study the response for a wide range of GR values, the only free parameter determining axon’s geometry (see Fig. 4G). Current pulses were induced at the upstream edge of the mother branch, and responses were measured along the two daughter branches.

Figure 4A–E show examples of firing patterns along branching points. Figure 4F is a phase plane diagram summarizing firing patterns as a function of stimulus frequency and GR, for intermediate frequencies (50–146 Hz). For frequencies lower than 50 Hz, propagation is determined solely by GR: propagates uninterruptedly if GR < 34.2 and fails otherwise. For frequencies higher than 146 Hz, failure of some spikes has already occurred in the mother branch before reaching the branching point as discussed above.

Seven distinct patterns of activities were identified; namely, trains, blockage, intermitted trains, single, several, complex and stuttering. These patterns were observed identically in the two daughter branches for different GR and frequency regimes as shown in Fig. 4F and are described below.

#### Trains

All spikes pass the branching point uninterruptedly (Fig. 4A).

#### Blockage

All spikes fail to pass (‘blocked’) the branching point.

#### Intermitted trains

Several consecutive spikes pass the branching point followed by a single failure. This definition also includes cases where a single spike passes the branching point successfully followed by another spike that fails (indicated as ‘1:1’ in Fig. 4F). An example of this behavior can be seen in Fig. 4B that shows a ‘3:1’ pattern. The spatial dynamics along the axon that leads to this pattern is shown in Supplementary Fig. S3, where three full amplitude spikes pass the branching point and continue to propagate along the daughter branches. The successive forth spike does not fully pass the branching point. Instead, only a low amplitude ‘hump’ passes, and decays passively along the daughter branches. As opposed to fragmented trains appeared in unbranched axon, in intermitted trains spike intervals are not equal.

#### Single

Only the first spike of the train is transmitted into the daughter branches (Fig. 4C). The spatial dynamics along the axon that lead to this pattern is shown in Supplementary Fig. S4.

#### Several

Similar to the single pattern but more than one spike succeed to propagate into the daughter branches.

#### Complex

A single spike followed by several failures (e.g., ‘1:2’, ‘1:3’, and ‘1:4’ patterns), or alternations between two intermitted train patterns, such as ‘3:1’ and ‘4:1’ that form ‘3:1:4:1’ pattern (Fig. 4D). This pattern is periodic.

#### Stuttering

Spike bursts separated by irregular quiescent intervals (Fig. 4E). An example of stuttering for relative long time is presented in Supplementary Fig. S5.

All the above behaviors can play different roles in signal modulation. The propagation of signals at different intermediate frequencies in branched axon is modulated symmetrically according to the GR value along the two daughter branches. For higher frequencies modulations may occur already at the unbranched segment level. Combined with the branching point modulations, asymmetric response along the daughter branches can emerge as can be seen in next section.

### Responses in asymmetric branched axons

Until now, we have analyzed symmetric branched axons, and measured symmetric responses along the two daughter branches. Most of the published literature that studied branched axons used GR as the single geometrical parameter that determines activity coding in branching points and reported symmetric responses. In line with previous simulation studies^17^, we have found that the responses are identical also in daughter branches with different radii, as long as the GR is the same. Supplementary Fig. S6 shows response pattern phase plane diagram for stimulus frequency and GR for non-equal radius daughter branches, compared to equal radius setting presented in Fig. 4F. Real branched axons, however, are usually not symmetric, and asymmetric activity was measured experimentally for high frequencies^44^.

**Figure 5:**
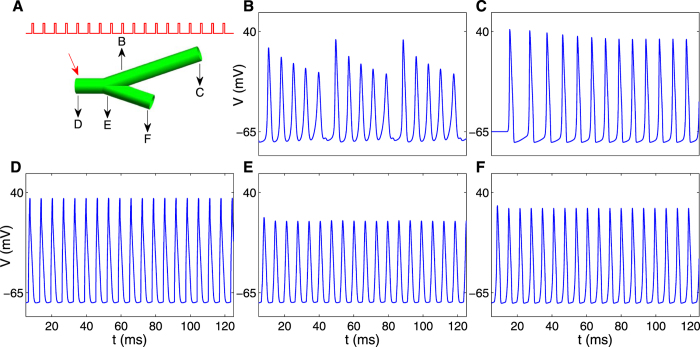
Example response to current stimuli along an asymmetric branched axon. (**A**) A schematic diagram of an asymmetric branched axon composed of daughter branches with identical radii and different lengths. Red arrow indicates the current stimulation point. Stimulus signal is represented by red line (154 Hz). (**B**–**F**) show the propagating signal dynamics measured in the points indicated by black arrows in (**A**), D at the stimulus point, B and C on the long branch 0.3 cm and 3 cm downstream of the branching point respectively, and E and F on the short branch 0.01 cm and 0.1 cm downstream of the branching point respectively. The system was set with a mother branch radius of 10 µm, GR of 1, and mother and daughter branch lengths of 0.1 cm, 0.1 cm, and 3 cm respectively.

Here we show how asymmetric activity can emerge from the results of the two previous sections. By concatenating an axonal segment to the edge of one of the daughters of a symmetric branching point we constructed new branched axon with identical GR value. Stimulating this new axon with high frequency current spikes resulted in asymmetric response along the two daughter branches. Figure 5 shows an example of this setup where response to high frequency stimulus is measured along an asymmetrical branching point. Asymmetry was constructed by setting different lengths for the two daughter branches, but maintaining identical radii. In this example, the trunk is relatively short (0.1 cm), enabling propagation of high frequency spike trains (154 Hz) from the beginning of the trunk (Fig. 5D) until the branching point. One of the daughter branches is longer than its sibling branch (Fig. 5A). All spikes of the train propagate along the shorter branch (Fig. 5E), while in the longer branch each sixth spike fails (Fig. 5B). Firing patterns (‘5:1’ at the long branch and ‘train’ at the short branch) can be inferred from unbranched axonal segments shown in Fig. 2F and H. Consequently, the frequency along the longer branch decreases to 128 Hz (Fig. 5C), while the train frequency along the shorter branch remains 154 Hz (Fig. 5F).

With this simple setup constructed from a symmetric branched axon concatenated to a linear unbranched segment we were able to show how asymmetric response emerged using high frequency stimulation. More generally, this example shows how understanding responses in unbranched axonal segments, and symmetric branching points enables us to understand behaviors of more realistic complex axonal trees.

## Discussion

The role of neuronal geometry in brain activity is instrumental. Here, we studied the two basic units of neuronal branching trees, axonal linear segments and axonal branching points. Gaining insights on the functionalities of these two fundamental motifs is crucial for better understanding signal propagation in more complex axonal structures. Here, we have used the classic Hodgkin Huxley model that was originally developed for studying electrophysiological dynamics in the squid giant axon. As such, the corresponding geometrical parameters used by the model are relatively large. Nevertheless, insights gained from the model showed relevance to other invertebrate and vertebrate systems which are smaller.

Starting with a linear axonal segment, we classified the response type as a function of stimulation frequency, radius and segment length. The response to high frequency stimulation demonstrates the influence of axonal geometry on activity patterns. Spike failures occur close to the upstream stimulation point. Farther along the axon, the surviving spikes shift in time to yield a lower frequency train pattern with equivalent intervals between spikes, known as ‘frequency-smoothing’ effect^55, 56^. We observed that trains propagate uninterruptedly until a critical length, where failures begin. A phenomenological equation of this length as a function of axon radius and train frequency was derived, and can be compared to other length scales from the literature. Rall in ref. 57 calculated a decay length scale (length constant, λ) for passive membrane, and Miller and Rinzel in ref. 55 calculated a length constant for an active membrane. Both expressions are proportional to the square root of the axonal radius, and do not depend on frequency. Eisenberg and Johnson in ref. 58 and Koch in ref. 59 studied quasi-active membranes, where axons are stimulated with sinusoidal signals using linear dynamics approximation. They derived an expression for the length constant that is inversely proportional to the square root of the frequency. Our expression for the critical length resembles the above functional dependencies (on radius and frequency), with adjustments due to the non-linearity considerations.

Moving from linear unbranched to branched axonal segments, where mother branch bifurcates into two daughter branches, led to a wealth of activity response patterns, ranging from regular intermittent trains to stuttering irregular patterns. This complex behavior repertoire starts at lower frequencies than for the linear segments, and demonstrates the ability to code information already at a single cell level. These intermediate frequency response patterns depend on GR and frequency only, and not on the scale of daughter branches radii, in line with^13, 17^. Even when daughter branches radii differ significantly, the two branches show identical response. When one of the daughter branches is shorter, the response along the two daughter branches remains the same.

At higher frequencies the effects of branching and daughter branches geometry (treated independently as linear unbranched segments) are combined. In branched axons, where sufficiently short trunks bifurcate into short and long daughter branches, non-symmetric responses were observed. High frequency trains passed uninterruptedly into the short daughter branches, but in the long sibling branches failures occurred, leading to fragmented train patterns. These failures may already occur at the end of the mother branches, due to influence from the daughter branches, in accordance with experimental observations^43^. Notably, this non symmetric response occurs even for GR of 1 (see Fig. 5), as has been observed experimentally in ref. 44. All results presented in this paper were obtained from a periodic stimulation of the axons. Concatenating axonal elements may result in non-periodic signals that stimulate down-stream axonal elements. Thus, examining the effects of non-periodic stimulation can help in understanding more complex geometrical structures of dendritic trees.

Previous theoretical studies predicted symmetrical responses in the two daughter branches (but see ref. 60), however, a number of experimental studies have demonstrated non symmetric conductions^42–46^. Explanations for these patterns were suggested by proposing external factors, such as a high axial resistivity at one daughter branch, ion concentration fluctuations, and external noise^17, 61, 62^. Here, we show how these non-symmetric conductions could result from geometrical properties alone.

The connections we show between the axonal tree structure, frequency and response patterns illustrate ways in which information is coded in the brain. Traditionally, axons were treated as simple cable elements, where spikes that failed to propagate were considered as an axonal dysfunction. Recently, the rich dynamics of signal propagation along axons have been interpreted as a possible mechanism for information coding^7^. These findings suggest that in addition to wiring optimization^63–66^ and energy consumption^67^, information coding considerations may drive neuronal structure.

In recent years, as computational and imaging techniques progress, neuronal morphological features were measured and made accessible through publically available large data repositories such as NeuroMorpho.Org^68^ and the Blue Brain Project^69, 70^. This detailed morphometric description of cells, together with understanding how geometry determines information flow, can open the possibility to deduce functionality from anatomical data.

## Methods

### Model setup

The HH spatially extended model was used for studying action potential propagation along unbranched and branched axons^71^. The following four nonlinear differential equations were used to study axonal response dynamics:

3 $$\begin{array}{rcl}\frac{a}{2R}\frac{{\partial }^{2}V}{\partial {x}^{2}} & = & {C}_{m}\frac{\partial V}{\partial t}+\overline{{g}_{K}}{n}^{4}(V-{V}_{K})+\overline{{g}_{Na}}{m}^{3}h(V-{V}_{Na})+\overline{{g}_{l}}(V-{V}_{l})-{I}_{ext}(x,t)\\ \quad \,\,\,\,\,\frac{\partial n}{\partial t} & = & {\alpha }_{n}(1-n)-{\beta }_{n}n\\ \quad \,\,\,\,\,\frac{\partial m}{\partial t} & = & {\alpha }_{m}(1-m)-{\beta }_{m}m\\ \quad \,\,\,\,\,\frac{\partial h}{\partial t} & = & {\alpha }_{h}(1-h)-{\beta }_{h}h\end{array}$$

where *V* is the membrane potential, and *m, h* and *n* are measures of sodium activation, sodium inactivation, and potassium activation, respectively. The current injected into the membrane is *I*_ext_. *α*_*m/h/n*_ and *β*_*m/h/n*_ represent the corresponding rates of gates opening and closing. Supplementary Table S1 summarizes all parameters of the model, and the equations that determine *α*_*m/h/n*_ and *β*_*m/h/n*_.

Matlab was used to simulate the system applying Crank-Nicolson method^72^. Negative voltage convention was used to set the resting potential to −65 mV. Length and time intervals were set to Δx = 100 µm, and Δt = 1 µs, respectively. We chose small enough segments to ensure numerical methods validity also for cases where an axon with a small diameter bifurcates into two branches with large diameters. Supplementary Fig. S7 shows the numerical results for two different length interval sizes, verifying the result in a specific case.

Two geometrical setups were studied, a simple straight axon, and a mother branch that bifurcates into two daughter branches (Fig. 1). All segments were set to be homogenous cylinders with boundary conditions of sealed ends^13, 73, 74^.

Current rectangular wave was generated in a particular frequency, at the edge of the axon to trigger neuronal activity of action potential propagating trains. Pulse width and amplitude were set to 1 ms and 15 mA/cm^2^, respectively.

## Electronic supplementary material

Supplementary information
