# Supplementary material for: Branching morphology determines signal propagation dynamics in neurons
Source: Sci Rep. 2017 Aug 21;7:8877. doi: 10.1038/s41598-017-09184-3 (PMC5567046; doi:10.1038/s41598-017-09184-3)
Supplement: Supplementary file 1 — Supplementary information [file 41598_2017_9184_MOESM1_ESM.pdf]

Branching morphology determines signal propagation dynamics in neurons

Supporting Information

Netanel Ofer[1,2], Orit Shefi[1,2], and Gur Yaari[1]

[1] *Faculty of Engineering, Bar Ilan University, Ramat Gan 5290002, Israel*
[2] *Institute for Nanotechnology and Advanced Materials, Bar Ilan University, Ramat Gan 5290002, Israel*

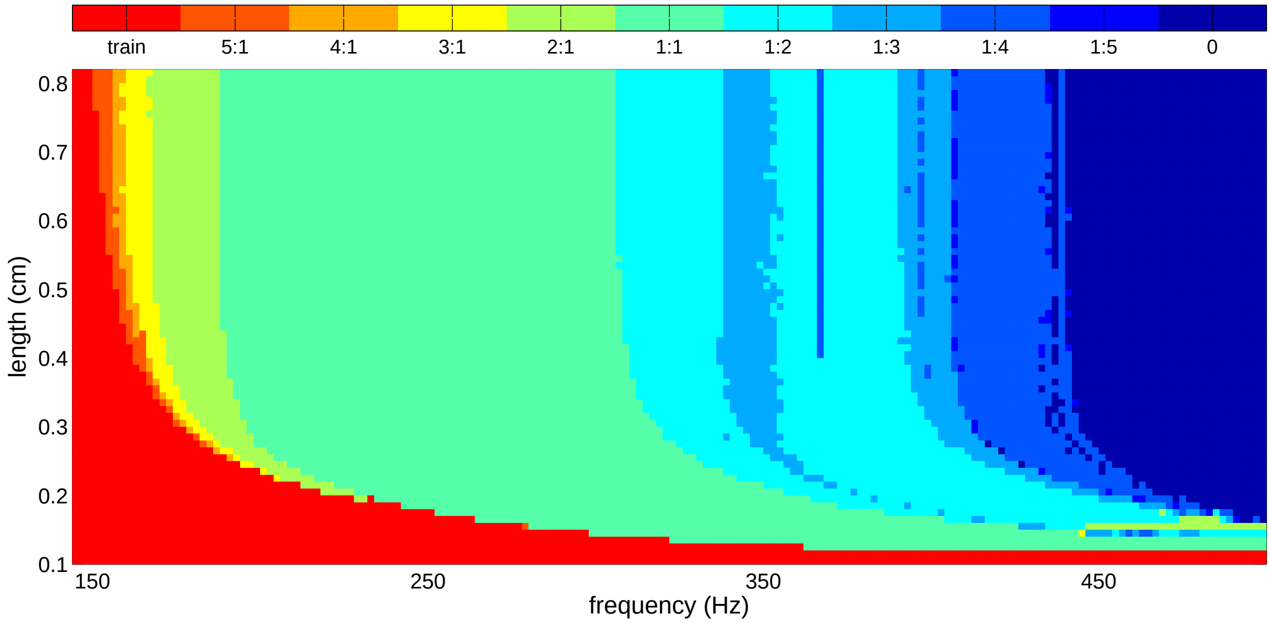

Figure S1: **Extended phase plane of the responses to current stimuli in unbranched axons.** Same as Fig. 2F for extended range of frequencies. The colors in the phase plane represent different firing patterns as shown in the color key. Simulation setup and parameters are the same as in Fig. 2F.

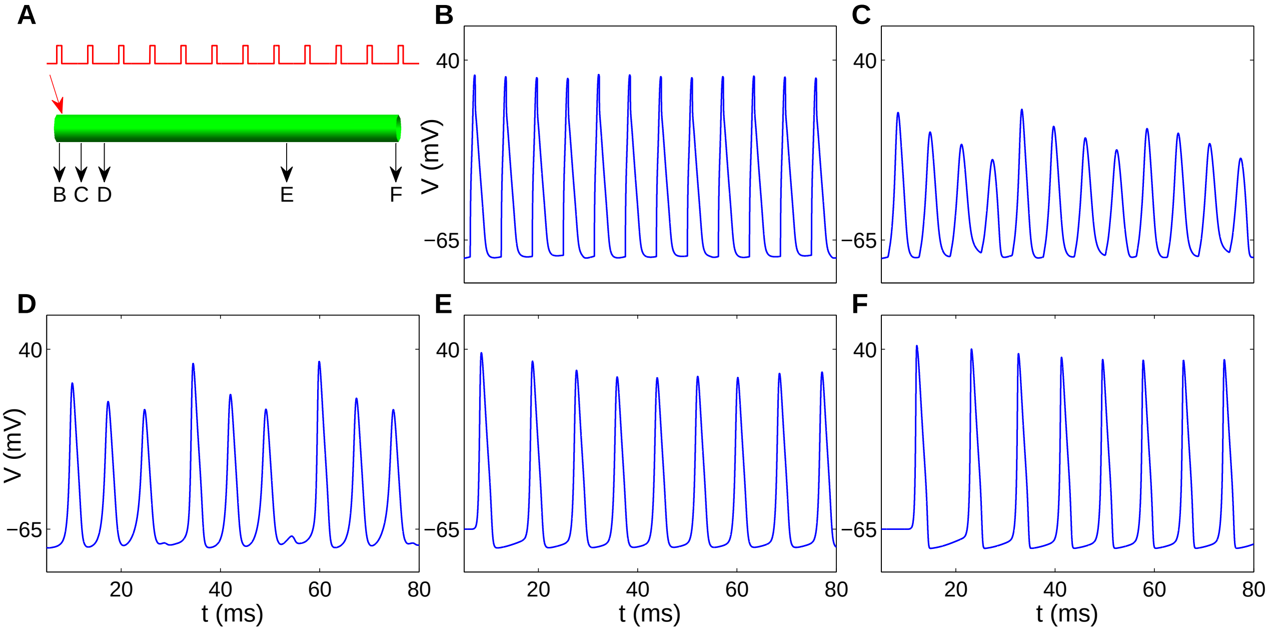

Figure S2: **'Fragmented train' pattern generation along a straight axon.** Each fourth spike fails to propagate along the axon, forming a pattern of '3:1', which leads to train frequency modulation. (A) A schematic diagram of a straight axon. Red arrow indicates the current stimulation point. Stimulus signal is illustrated by a red line $(160Hz)$. (B-F) Signal propagation dynamics measured in the points indicated by black arrows in (A), at the stimulus point, $0.15cm$, $0.39cm$, $2cm$, and $3cm$ along the axon. Axon radius was set to $10\mu m$, and axon length to $3cm$.

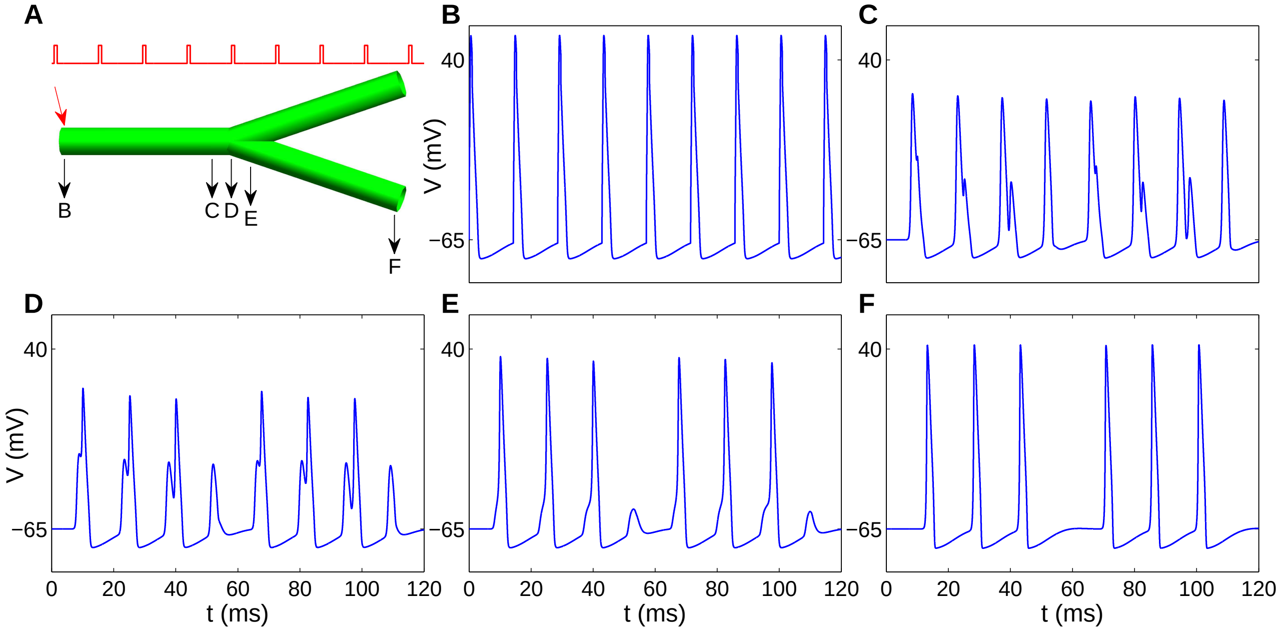

Figure S3: **Example response of 'intermitted trains'.** (A) A schematic diagram of a symmetric branched axon. Red arrow indicates the current stimulation point. Stimulus signal is illustrated by a red line $(70Hz)$. (B-F) Signal propagation dynamics measured in the points indicated by black arrows in (A), at the stimulus point, $0.04cm$ before branching point, $0.01cm$ before branching point, $0.01cm$ after branching point, and at the end of the daughter branch. The '3:1' pattern is kept along the daughter branch, even for long distances (F). The radius of the mother branch was set to $10\mu m$, GR to 21, and mother and daughter branch lengths to $2cm$.

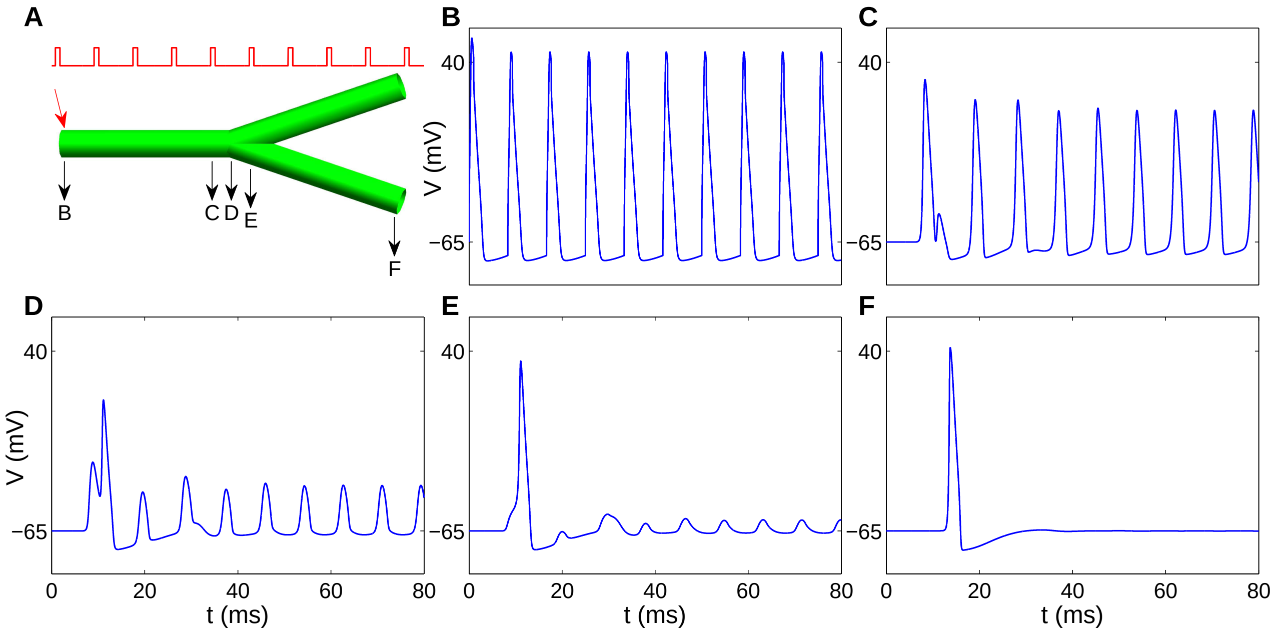

Figure S4: **Example response of a 'single' pattern.** (A) A schematic diagram of a symmetric branched axon. Red arrow indicates the current stimulation point. Stimulus signal is illustrated by a red line $(120Hz)$. (B-F) Signal propagation dynamics measured in the points indicated by black arrows in (A), at the stimulus point, $0.06cm$ before branching point, $0.01cm$ before branching point, $0.01cm$ after branching point, and at the end of the daughter branch. Mother branch radius was set to $10\mu m$, GR to 30, and mother and daughter branch lengths to $2cm$.

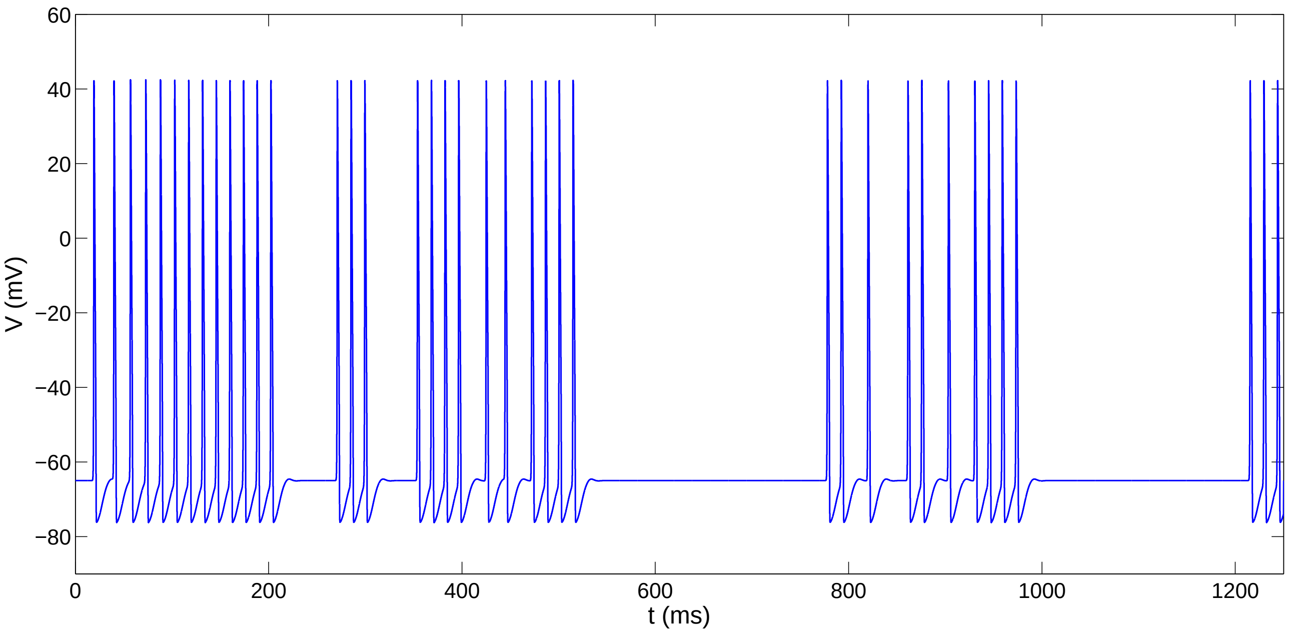

Figure S5: **Typical pattern of stuttering for relatively long time.** The response was measured at the end of the daughter branch of a branching point with GR = 15 as a response to stimulus of $144Hz$.

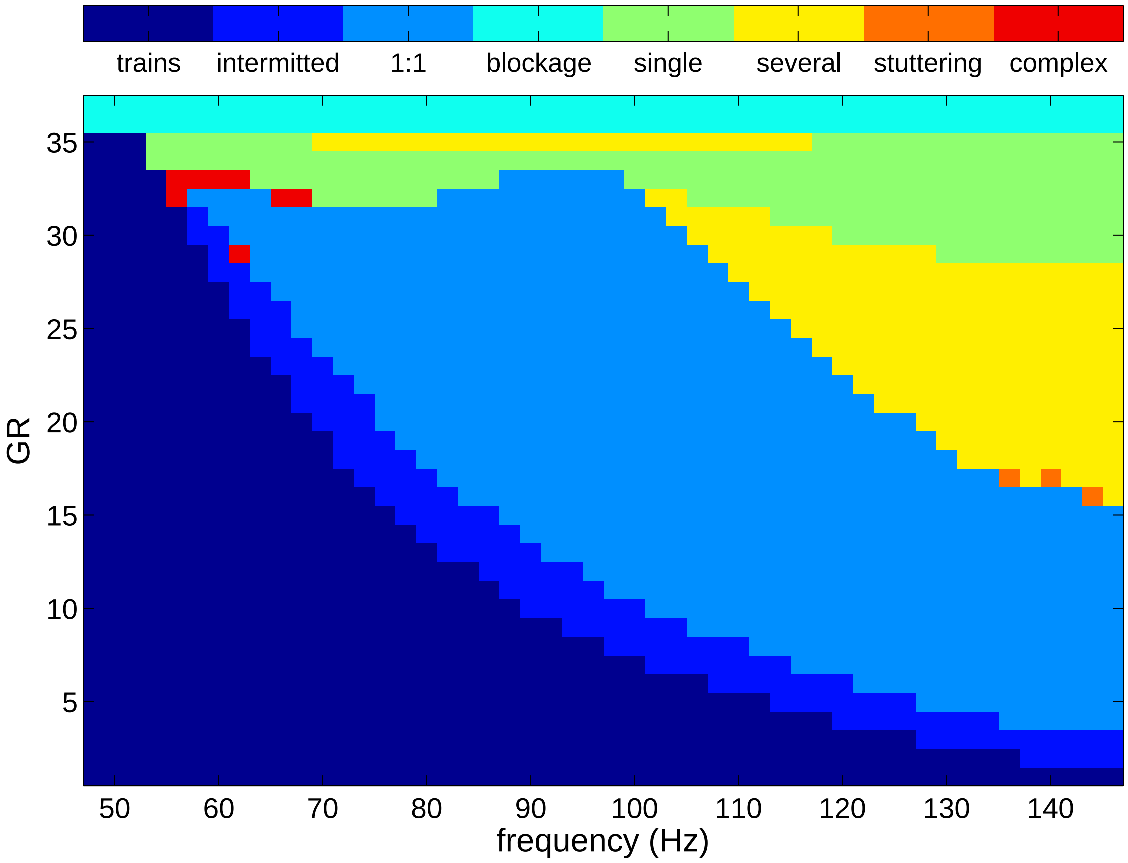

Figure S6: **Response pattern phase plane for non-equal daughter branches radii.** The radius of one of the branches was set to twice the radius of its sibling branch; compared to equal radius setting presented in Fig. 4F.

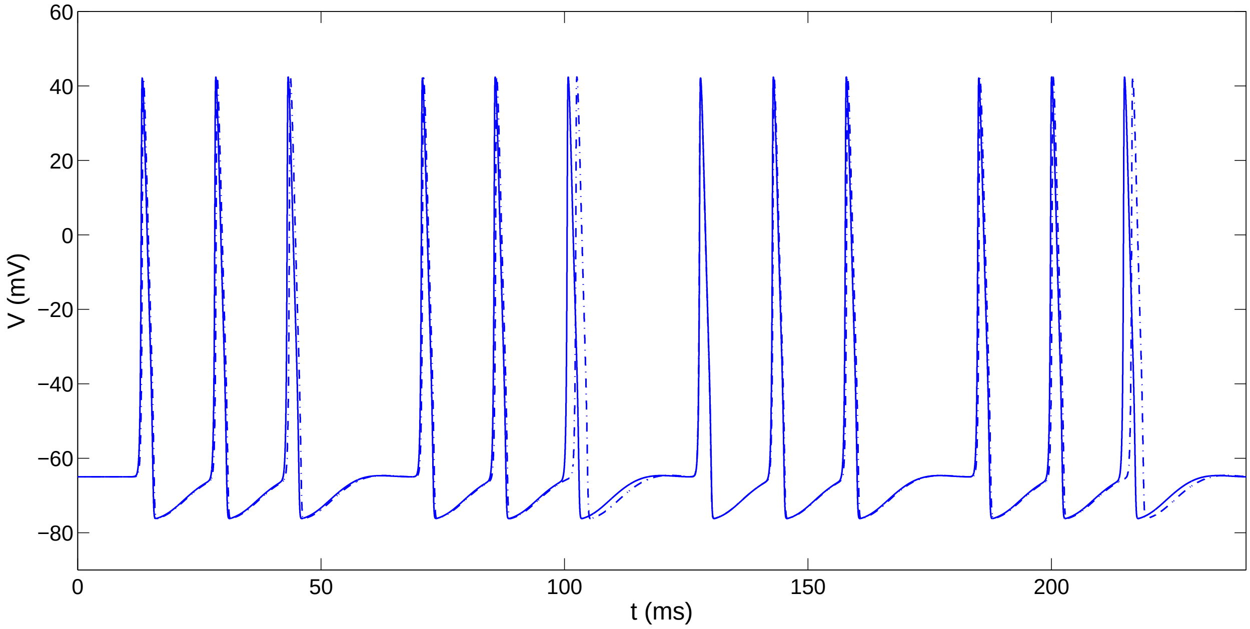

Figure S7: **Examination of numerical precision.** '3:1' intermitted pattern, GR = 21, frequency of $70Hz$, $\Delta x = 100\mu m$ (solid line), and $\Delta x = 50\mu m$ (dashed line).

Supplementary Note S1

Temperature effects

The neuronal membrane activity is influenced by temperature. As temperature increases, the kinematics of the ion channels is faster, allowing for higher train frequencies to propagate. To study the effect of temperature on the results, we repeat our study in higher temperature of 20°C.

Parameter estimation of the phenomenological equation that describes the transition between propagating train and coded firing patterns for temperature of 20°C is presented in Fig. S8. Equation (1) shows the derived equation, the form of the equation remains the same, except that the coefficient (γ) increases, meaning that the spike failures occur at longer axons.

$$l_c(a,f) = 0.69\frac{a^{0.465}}{(f - f_c(a))^{0.45}} \tag{S1}$$

In 20°C the range of the 'intermediate frequencies' in branched axon was between 180 and 450 Hz compared to 50-146 Hz in 6.3°C. Frequencies higher than 450Hz are considered 'high frequencies' and may lead to spike failures even along unbranched axons. In addition, the critical GR decreases as temperature increases. The critical GR value above which spikes fail to cross the branching point was 10.8, compared to 34.2 in 6.3°C.

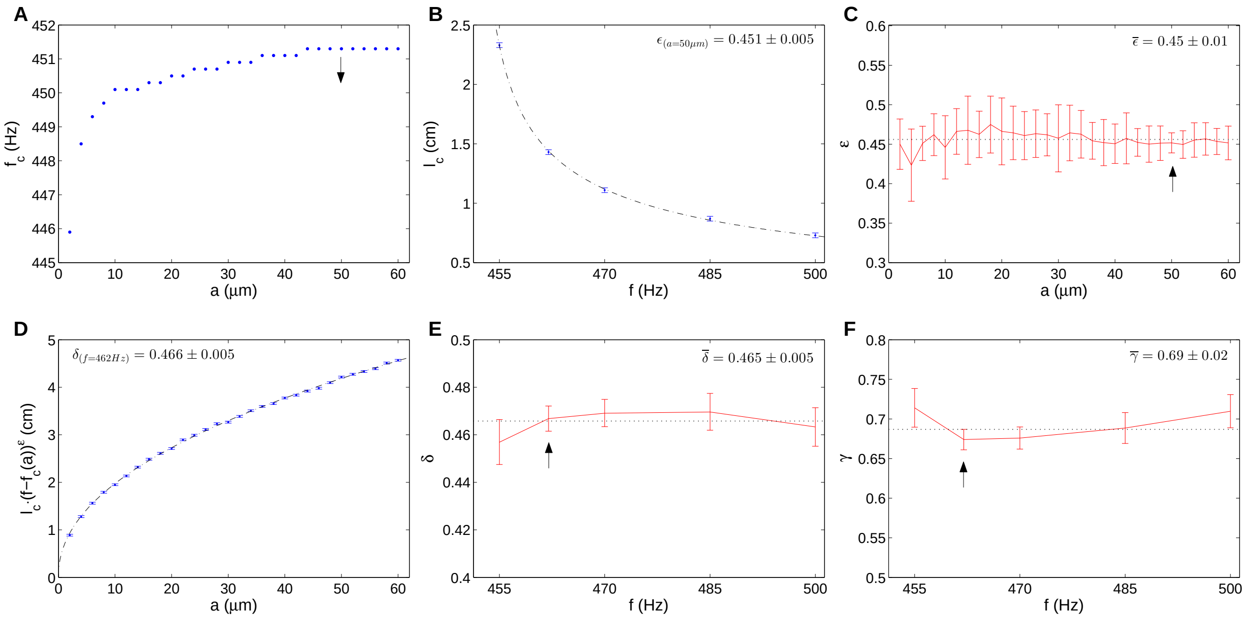

Figure S8: **Parameter estimation of the phenomenological equation that describes the transition between propagating train and coded firing patterns for temperature of 20°C.** (A) Direct measurements of the maximum frequency (f_c) that enables propagation of uninterrupted train in long axons $(10cm)$ for a range of axonal radii. Arrow indicates radius value used in (B). (B) Fitting the dependency between l_c and f for $a = 50\mu m$. Dashed line is the fitted curve: $4.199(f - 451.3)^{-0.451}$. Bars correspond to numerical measurement errors determined by the sampling interval of the phase plain: $\Delta l = 0.02cm$. (C) Fitted ϵ's for all studied radii. Arrow indicates ϵ value calculated in (B). (D) Fitting the dependency between $l_c(f - f_c(a))^\epsilon$ and a for $f = 462Hz$. Dashed line is the fitted curve: $0.674(f - f_c(a))^{-\epsilon}a^{0.466}$. Bars correspond to numerical measurement errors determined by the sampling interval of the phase plain: $\Delta l = 0.02cm$. (E-F) Fitted δ's (E) and γ's (F) for all studied frequencies. Arrows indicate the δ and γ values calculated in (D).

Supplementary Table S1

Hodgkin Huxley simulation parameters

Parameters	Description	Values	Units
C	Membrane capacitance	1	$\mu F/cm^2$
$\overline{g}_K$	Maximal potassium conductance	36	mS/cm^2
V_K	Potassium reversal potential	-77	mV
$\overline{g}_{Na}$	Maximal sodium conductance	120	mS/cm^2
V_{Na}	Sodium reversal potential	50	mV
g_L	Leak conductance	0.3	mS/cm^2
V_L	Leak reversal potential	-54.401	mV
R	The axoplasm specific resistance	35.4	Ωcm
a	The axonal radius	238	μm
T	Temperature	6.3*	$^\circ C$

* We used $T = 6.3^\circ C$ as in the original Hodgkin Huxley paper [1]. Changes in temperature influence the gating variables α's and β's by multiplying them by $\phi = 3^{(T-6.3)/10}$.

Hodgkin Huxley Equations

$$\alpha_n = \frac{0.01(V+10)}{e^{\frac{(V+10)}{10}} - 1} \tag{S2}$$

$$\beta_n = 0.125 e^{\frac{V}{80}} \tag{S3}$$

$$\alpha_m = \frac{0.1(V+25)}{e^{\frac{(V+25)}{10}} - 1} \tag{S4}$$

$$\beta_m = 4 e^{\frac{V}{18}} \tag{S5}$$

$$\alpha_h = 0.07 e^{\frac{V}{20}} \tag{S6}$$

$$\beta_h = \frac{1}{e^{\frac{(V+30)}{10}} + 1} \tag{S7}$$

1. Hodgkin, A. L., Huxley, A. F. A quantitative description of membrane current and its application to conduction and excitation in nerve. *J Physiol* **117**, 500544 (1952).
